# Tuberculosis Examination Methods in the Army

**Published:** 1919

**Authors:** 


***Tuberculosis Examination Methods in the Army***. By I. S. Kahn. *Southern Medical Journal,* November 1918.

By far the larger number of tuberculous cases encountered in military chest examinations present absolutely no clinical evidences of their disease beyond slight cough.

Tuberculous infection may result in general military septicemia, or may grade down to the usual manifestations of a disease of prolonged chronicity of several years' duration before a fatal termination, or of a prolonged chronicity never leading to death, or may finally be checked after considerable involvement.

The case of tuberculosis with noticeable cough and clinical toxic symptoms of a few weeks' duration may not be an early case at all, but may present more or less extensive consolidation of months' or years' duration, all the while possessing fine inspiratory rales following cough, indicating disease activity not previously recognized because of lack of cause for the search of its presence.

In determining the diagnosis of active pulmonary tuberculosis by physical signs, the classical admitted evidences of such activity are represented by unilateral lagging, deficient expansion, the presence of dulness, modified voice and respiratory sounds, and rales usually crepitant and subcrepitant, later becoming coarse or more musical in character in the presence of caseation and cavity formation.

Pulmonary consolidations, even if only small or of multiple extent, may be due to causal organisms other than the tubercle bacillus. Often the chest findings of an acute respiratory infection will simulate perfectly the findings of tuberculous consolidation. Fine crepitant rales are often due to postpneumonic, pleuritic adhesions. It is essential that judgment of a case be not made on a single examination. Extensive physical signs may exist with very slight clinical evidence.

In this work, the authors find the fluoroscope of considerable value. In a high percentage of cases, its findings have given immediate confirmation, especially in the more gross lesions. However, in far less than 1 o/o of cases have the authors been unable to secure satisfactory radiographic proof of the physical findings.

The army regulations set as a standard of rejection the presence of inactive lesions of more than appreciable size below the apex.

In order to facilitate the work at Camp MacArthur, a board consisting of from 12 to 8 examiners was divided into two groups: 1. The routine examining board, consisting of from 8 to 9 examiners. 2. The consulting board, consisting of the three most experienced members.

When a routine examiner encounters a case of chest abnormality of any kind whatsoever, such a case is recorded on a card upon which the examiner notes briefly the abnormal or suspected findings. The abnormal cases, by a filing system, return automatically to the original examiner at the end of a week. At this second examination, the examiner spends as much time as he deems necessary clearing such cases as he can, and holding doubtful cases for further examination.

The consulting section of the board sits in constant session and acts upon all cases referred by the routine examiners.

It will thus be seen that the army tuberculosis examination is not a perfunctory affair, and that conclusions are reached only after mature consideration of every case on its merits, at times covering an observation period of many weeks.

